# Controlled Synthesis of Monodisperse Hexagonal NaYF_4_:Yb/Er Nanocrystals with Ultrasmall Size and Enhanced Upconversion Luminescence

**DOI:** 10.3390/molecules22122113

**Published:** 2017-12-01

**Authors:** Hui Li, Lei Xu, Guanying Chen

**Affiliations:** MIIT Key Laboratory of Critical Materials Technology for New Energy Conversion and Storage, School of Chemistry and Chemical Engineering & Key Laboratory of Micro-Systems and Micro-Structures, Ministry of Education, Harbin Institute of Technology, Harbin 150001, China; huili@hit.edu.cn (H.L.); xulei82@hit.edu.cn (L.X.)

**Keywords:** ultrasmall size, optimum concentration, upconversion luminescence, core–shell structure

## Abstract

The ability to synthesize upconversion nanocrystals (UCNCs) with tailored upconversion luminescence and controlled size is of great importance for biophotonic applications. However, until now, limited success has been met to prepare bright, ultrasmall, and monodispersed β-NaYF_4_:Yb^3+^/Er^3+^ UCNCs. In this work, we report on a synthetic method to produce monodisperse hexagonal NaYF_4_:Yb^3+^/Er^3+^ nanocrystals of ultrasmall size (5.4 nm) through a precise control of the reaction temperature and the ratio of Na^+^/Ln^3+^/F^−^. We determined the optimum activator concentration of Er^3+^ to be 6.5 mol % for these UCNCs, yielding about a 5-fold higher upconversion luminescence (UCL) intensity than the commonly used formula of NaYF_4_:30% Yb^3+^/2% Er^3+^. Moreover, a thin epitaxial shell (thickness, 1.9 nm) of NaLnF_4_ (Ln = Y, Gd, Lu) was grown onto these ultrasmall NaYF_4_:Yb^3+^/Er^3+^ NCs, enhancing its UCL by about 85-, 70- and 50-fold, respectively. The achieved sub-10-nm core and core–shell hexagonal NaYF_4_:Yb^3+^/Er^3+^ UCNCs with enhanced UCL have strong potential applications in bioapplications such as bioimaging and biosensing.

## 1. Introduction

Lanthanide-doped upconversion nanocrystals (UCNCs) are able to produce strong anti-Stokes luminescence, in which two or more low energy photons are stepwise absorbed via real intermediate long-lived electronic states of lanthanides, resulting in an excitation of a higher electronic state that produces an emission of higher energy photon [1]. They have superior physiochemical characteristics, such as excellent thermal and chemical stability, non-photobleaching, non-blinking, sharp emission bandwidths, low toxicity, and large anti-Stoke shift to entail zero optical imaging background. These features make UCNCs suitable for a broad spectrum of potential applications ranging from three-dimensional displays [2], to high contrast small animal imaging [3,4,5,6], and to high sensitivity bio-detection [7,8].

Among reported UCNCs, hexagonal phase (β-) sodium yttrium fluoride (NaYF_4_) has been shown to be one of the most efficient upconverting host materials, owing to its low phonon cutoff energy of ~350 cm^−1^ that is able to effectively reduce non-radiative energy losses at the intermediate states of lanthanide ions. As a result, lanthanide-doped β-NaYF_4_ UCNCs with controlled size and shape have gained a great deal of interest and have been widely investigated for biomedical applications. In particular, sub-10-nm (ultrasmall) ones are preferable in this regard, as this size is comparable to that of large proteins and thus allows a rapid clearance of nanoparticles from the body after completing their intended roles [9,10,11]. The uniform β-NaYF_4_:Yb^3+^/Er^3+^ nanoparticles >20 nm in size have been successfully prepared at high temperature (300–320 °C) by many groups [12,13,14,15]. Meanwhile, attempts for the preparation of ultrasmall hexagonal NaLnF_4_ (e.g., NaYF_4_) nanoparticles have been made through a decrease in reaction temperature (200–280 °C) or reaction time (shorter than 20 min) [16,17]. Yet, since the β-phase is thermodynamically less stable than the α-phase (cubic phase), the high surface tension of small-sized nanocrystals usually leads to a phase transformation from anisotropic (β) to isotropic (α), thus resulting in the production of nanoparticles of mixed crystallographic phases. Doping the host matrix with a high concentration of lanthanide ions with ionic radii larger than Y^3+^, such as gadolinium (Gd) [18,19], europium (Eu), and terbium (Tb) [20] can help stabilize the β-phase, but such doping will alter the optical properties of NaYF_4_-based upconverting nanoparticles. Few works have reported the successful synthesis of monodisperse sub-10-nm β-NaYF_4_ particles that are free from the heavy doping of lanthanide ions of larger ionic radii. Ostrowski et al. [21] achieved this by using the “cooperative effect” between oleylamine and oleic acid coordinating ligands via thermolysis of metallic trifloroacetates at a high temperature, while the Haase group [22] obtained sub-10-nm hexagonal NaYF_4_:Yb/Er UCNCs by increasing the Na:Y ratio using a co-precipitation method with critical reaction parameters. However, the former requires a stringent combination of both growth-controlling surfactant ligands with delicately defined reaction parameters, while the latter method requires the demanding use of purified α-phase particles to be firstly prepared and then utilized as the sacrificial nanoparticles to produce β-phase ones via Ostwald-ripening processes. Moreover, the UCL of these obtained sub-10-nm UCNCs is inadequate, needing a substantial improvement towards practical bioapplications.

Herein, we develop a synthetic method of preparing sub-10-nm β-NaYF_4_:Yb^3+^/Er^3+^ core and NaYF_4_:Yb^3+^/Er^3+^@NaYF_4_ core–shell UCNCs, and this method does not use α-phase particles as sacrificial particles. The crystallographic phases of the product can be easily controlled by altering reaction temperature, while the resulting particle size can be precisely tailored by the variation of the ratio of Na^+^/Ln^3+^/F^−^. Additionally, the optimized activator concentration of Er^3+^ ions for these ultrasmall NaYF_4_:30%Yb^3+^, *x*% Er^3+^ UCNCs was determined to be 6.5%, about 3 times higher than the commonly used formula of NaYF_4_:20–30% Yb^3+^/2% Er^3+^ for large-sized UCNCs (20–100 nm). Moreover, we prepared a set of sub-10-nm NaYF_4_:Yb^3+^/Er^3+^@NaYF_4_, NaYF_4_:Yb^3+^/Er^3+^@NaGdF_4_, and NaYF_4_:Yb^3+^/Er^3+^@NaLuF_4_ core–shell nanocrystals of varying shell host materials, and investigated the shell host effect on UCL intensity. We found that the UCL of the core nanoparticles was enhanced by a factor of 85-, 70- and 50, respectively, manifesting the highest UCL from the NaYF_4_:Yb^3+^/Er^3+^@NaYF_4_ core–shell UCNCs.

## 2. Results and Discussion

### 2.1. Control of the Phase and Size of NaYF4:Yb/Er Nanoparticles

**Figure 1:**
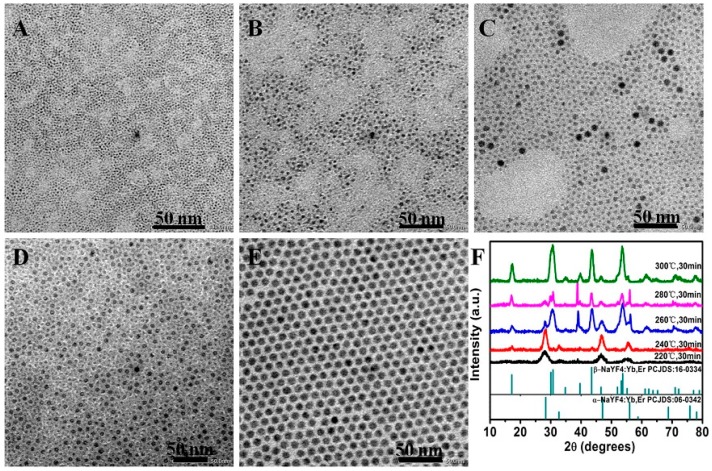
(**A**–**E**) TEM images of NaYF_4_:30% Yb^3+^/2% Er^3+^ obtained at different reaction temperature of 220, 240, 260, 280 and 300 °C for half an hour, respectively. The ratio of Na^+^/Ln^3+^/F^−^ equals 2.5:1:4; (**F**) The corresponding powder X-ray diffraction (XRD) patterns of the nanocrystals shown in (**A**–**E**). The standard diffraction patterns of the α-NaYF_4_ (JCPDS 06-0342) and the β-NaYF_4_ (JCPDS 16-0334) are displayed at the bottom for reference.

The crystal structure of NaYF_4_ typically consists of two forms, i.e., the cubic (α-) and the hexagonal (β-) phases. Since hexagonal NaYF_4_ UCNCs are able to offer about one order of magnitude higher UCL intensity than the cubic ones, we are interested in preparing ultrasmall NaYF_4_ UCNCs of β-phase [23,24]. In order to obtain small-sized β-NaYF_4_:30% Yb^3+^/2% Er^3+^, we prepared a set of nanocrystals using a co-precipitation method adapted from the literature [12], and explored the effect of reaction temperature on the crystal phase of the resulting nanoparticles. The nanoparticles were synthesized with a fixed ratio of Na^+^/Ln^3+^/F^−^ of 2.5:1:4 in the growth solution, but at varied reaction temperature of 220–300 °C. The corresponding TEM images and XRD patterns of the as-prepared nanoparticles are compiled and shown in Figure 1. As one can see, at a low temperature of 220 °C, the as-prepared NaYF_4_:30% Yb^3+^/2% Er^3+^ UCNCs are uniform in size with a spherical shape, whose XRD pattern is in line with the standard cubic one of JCPDS 06-0342 (Figure 1F). For a temperature between 240 and 280 °C, the diffraction peaks of β-NaYF_4_ begin to appear and coexist with that of α-NaYF_4_, which is in agreement with the observation of depolarization of particle size (Figure 1B–D). At 300 °C, pure hexagonal NaYF_4_:30% Yb^3+^/2% Er^3+^ nanocrystals with a uniform size of ~8 nm have been achieved, as shown in Figure 1A,F. This result indicates that a high temperature favors the synthesis of pure hexagonal phase nanocrystals of a lower symmetry.

**Figure 2:**
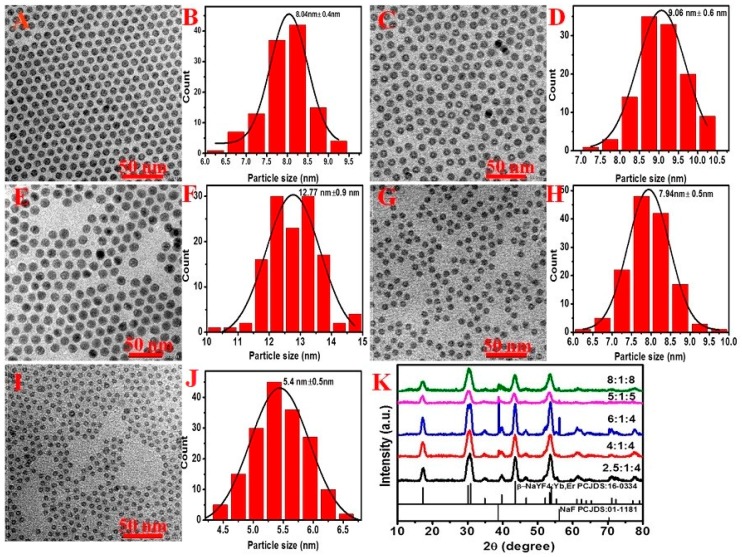
TEM images and size histograms of NaYF_4_:30% Yb/2% Er nanocrystals synthesized by using Na^+^, Ln^3+^, and F^−^ with a molar ratio of 2.5:1:4 (**A**,**B**), 4:1:4 (**C**,**D**), 6:1:4 (**E**,**F**), 5:1:5 (**G**,**H**), 8:1:8 (**I**,**J**), respectively. Reaction temperature: 300 °C. The corresponding powder X-ray diffraction (XRD) patterns of the nanocrystals are shown in (**K**); the standard diffraction patterns of the β-NaYF_4_ (JCPDS 16-0334) and NaF (JCPDS 01-1181) are depicted at the bottom for reference.

The formation of NaYF_4_:Yb^3+^/Er^3+^ nanocrystals typically includes two growth stages: nucleation and crystal growth. As the elemental nutrients of NaYF_4_ UCNCs, the amount of Na^+^ and F^−^ plays a significant role in defining the size of the resulting nanoparticle by shaping the nucleation process [25,26,27]. To investigate the concentration effect of precursors on the resulting particle size, we synthesized a range of NaYF_4_:30% Yb^3+^/2% Er^3+^ nanocrystals at 300 °C with different ratios of Na^+^/Ln^3+^/F^−^. The corresponding TEM images and XRD patterns of samples are shown in Figure 2. As one can see, when increasing the NaOA content from 2.5 to 4 mmol (corresponding to an increase of Na^+^/Ln^3+^/F^−^ from 2.5:1:4 to 4:1:4) and then to 6 mmol (corresponding to Na^+^/Ln^3+^/F^−^ of 6:1:4), a gradual narrowing of the main XRD peaks at 30.7° and 42° is observed, indicating an increase in the size of the final products according to the Scherrer’s equation [27]. Indeed, this observation is consistent with the acquired results of TEM and size histograms, whereby the size is shown to increase from 8.0 to 12.7 nm (Figure 2A–F). Moreover, in addition to the diffraction peaks of β-NaYF_4_, the diffraction peaks of NaF are also observed at the ratios of Na^+^/Ln^3+^/F^−^ of 4:1:4 and 6:1:4, being stronger for the latter with a higher dose of NaOA precursor. This result implies that an excessive NaOA content favors the formation of NaF byproduct, while the fluorine-deficiency in the growth solution leads to the formulation of large β-phase seeds, which accordingly mediate the growth of β-NaYF_4_:Yb/Er nanocrystals with large size. We reason that a simultaneous increase of Na^+^ and F^−^ content in the solution can result in the production of small β-phase seeds, thus enabling the growth of final UCNCs with a reduced size. Indeed, as expected, the size of nanocrystals was decreased from 9.1 to 7.9 nm when the molar ratio of Na^+^/Ln^3+^/F^−^ was elevated to be 5:1:5 (Figure 2G,H), and then to 5.4 nm when the molar ratio was further increased to 8:1:8 (Figure 2I–K). A simultaneous control of both the concentrations of NaOA and the NH_4_F precursors provides a simple method of producing monodisperse β-NaYF_4_:Yb^3+^/Er^3+^ nanocrystals with tailored sub-10-nm size, without the necessity of altering reaction time and temperature, as commonly employed in literature works [16,17]. In addition, due to surface-related quenching effects, the UCL intensity decreases with the decrement of particle size (Figure S1).

### 2.2. Enhancing Upconversion Luminescence of Ultrasmall β-NaYF_4_:Yb/Er

**Figure 3:**
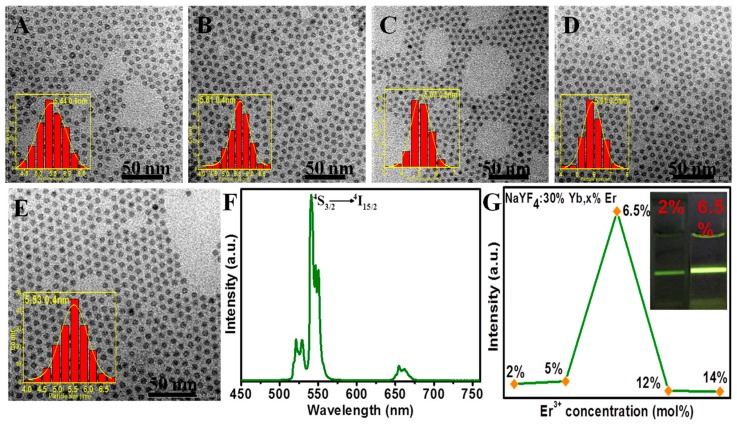
TEM images of NaYF_4_:30% Yb/*x*% Er (*x* = 2 (**A**), 5 (**B**), 6.5 (**C**), 12 (**D**), 14 (**E**)) nanocrystals prepared by using Na^+^, Ln^3+^, and F^−^ in a ratio of 8:1:8 and reaction temperature of 300 °C (reaction time: 30 min). The corresponding histograms of size distributions of NaYF_4_:30% Yb/*x*% Er are shown in the inset of (**A**–**E**); (**F**) UCL spectra in the spectral range of 450–750 m for NaYF_4_:30%Yb/2% Er UCNCs under 980 nm laser diode excitation; (**G**) The dependence of integrated UCL intensity from 500 to 600 nm on the Er^3+^ concentration in NaYF_4_: 30%Yb/*x*% Er UCNCs. The inset in (**G**) displays recorded digital photos of UCL from NaYF_4_:30% Yb/2% Er and NaYF_4_:30% Yb/6.5% Er UCNCs excited at 980 nm.

The concentration of lanthanide activator defines the number of emitting centers in the UCNCs to radiate the harvested excitation photonic energy, thereby dictating the overall UCL intensity of UCNCs. To explore the concentration effect of Er^3+^ on the resulting UCL, a series of β-NaYF_4_:30% Yb/*x*% Er UCNCs with varying concentrations of Er^3+^ (*x* = 2, 5, 6.5, 12, 14) were prepared at 300 °C for 30 min at a molar ratio of Na^+^/Ln^3+^/F^−^ of 8:1:8. Note that the concentrations of Er^3+^ of β-NaYF4:30% Yb/*x*% Er UCNCs have been calibrated by the results of inductive coupled plasma atomic emission spectroscopy (ICP-AES), as shown in Table S1. All resulting NaYF_4_:Yb^3+^/Er^3+^ nanocrystals exhibit almost identical particle size (about 5.4 nm) and narrow size distribution, as demonstrated in Figure 3A–E. Therefore, the effect of particle size on the UCL intensity can be ruled out. When excited at 980 nm, all samples dispersed in hexane emit a green UCL peaked at 520/540 nm and a red UCL peaked at 659 nm, which correspond to the ^2^H_11/2_/^4^S_3/2_→^4^I_15/2_ and ^4^F_9/2_→^4^I_15/2_ transitions of Er^3+^ ions, respectively (Figure 3F). The integrated intensity of the green peak (from 500 to 600 nm) as a function of Er^3+^ concentration is plotted in Figure 3G. As one can see, the UCL intensity increases first with an increase in Er^3+^ concentration, reaches the highest at the concentration of 6.5%, and then declines at higher Er^3+^ concentrations. Notably, the UCL intensity for nanocrystals at the optimized concentration of 6.5% is about fivefold higher than the one of β-NaYF_4_:30% Yb/2% Er, a commonly reported optimal formulation for large-sized UCNCs (≥20 nm). The different optimized activator concentration from the literature might be associated with the size effects, as ultrasmall nanocrystals have higher surface-related quenching effects and thus demand higher activator concentrations to reach a balance between the emitting process and the deactivation process. The decrease in UCL intensity beyond an Er^3+^ concentration of 6.5% can be ascribed to the well-known luminescence concentration quenching effect, which is prevalent for all types of reported luminophores.

**Figure 4:**
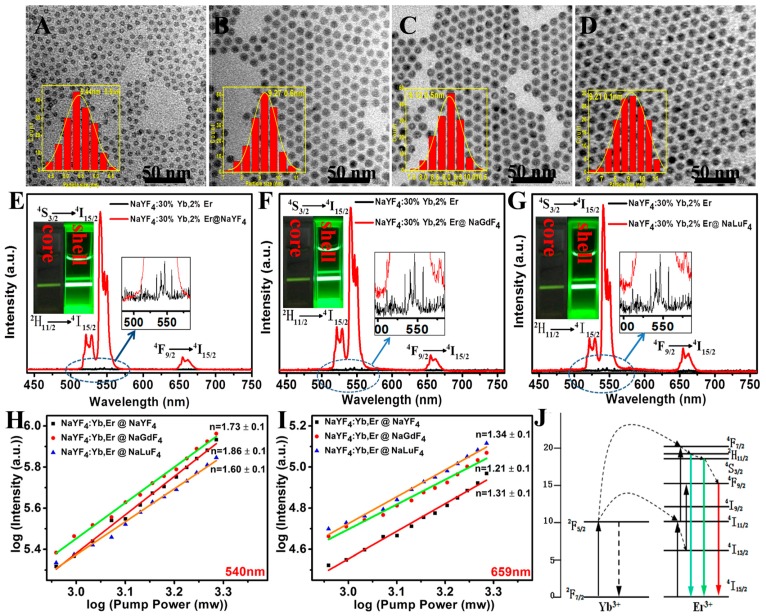
(**A**–**D**) TEM images and histograms of size distributions (inset) of NaYF_4_:30% Yb/6.5% Er, NaYF_4_:30% Yb/6.5% Er@NaYF_4_, NaYF_4_:30% Yb/6.5% Er@NaGdF_4_, NaYF_4_:30% Yb/6.5% Er@NaLuF_4_. All the core nanocrystals were prepared with the ratio of Na^+^/Ln^3+^/F^−^ is 8:1:8 at reaction temperature of 300 °C; (**E**–**G**) UCL spectra from the core NaYF_4_:30% Yb/6.5% Er and the core–shell NaYF_4_:30% Yb/6.5% Er@NaYF_4_, NaYF_4_:30% Yb/6.5% Er@NaGdF_4_, NaYF_4_:30% Yb/6.5% Er@NaLuF_4_ UCNCs (hexane dispersion). The inset shows the corresponding UCL photographic images. Excitation at 980 nm, 100 W/cm^2^. Dependencies of emission band intensities at 540 nm (**H**) and 659 nm (**I**) on the incident laser pump power; (**J**) Schematic illustration of the proposed upconversion pathways between Yb^3+^ and Er^3+^ ions.

To further enhance the UCL intensity of ultrasmall β-NaYF_4_:Yb/Er nanocrystals with an optimized Er^3+^ concentration, we prepared a set of core–shell nanoparticles with a different shell host lattice, which consisted of a β-NaYF_4_:30% Yb/6.5% Er core and a shell of NaYF_4_, NaGdF_4_, or NaLuF_4_. TEM images show that the particle sizes of all as-prepared core–shell nanocrystals are identical at about 9.2 nm (Figure 4A–D). This suggests that the NaLnF_4_ (Ln = Y, Gd, Lu) shell with a thickness of 1.9 nm was successfully coated onto the surface of β-NaYF_4_:Yb/Er core nanocrystals (size: 5.4 nm). The contrasted UCL spectra from the NaYF_4_:30% Yb/6.5% Er core and the NaYF_4_:30% Yb/6.5% Er@NaLnF_4_ (Ln = Y, Gd, Lu) core–shell nanocrystals are shown in Figure 4E–G. Though the spectral shape for both the core and the core–shell UCNCs is identical, the UCL intensity is substantially different, as all the core–shell samples have a significantly higher intensity than the core nanoparticles. According to Figure 4E–G, the UCL intensity from the NaYF_4_:30% Yb/6.5% Er@NaLnF_4_ (Ln = Y, Gd, Lu) core–shell UCNCs was calculated to be about 85-, 70- and 50-fold higher than that of the core nanocrystals, respectively. The significant enhancement can be also easily discerned from photographic UCL images of the core and the core–shell UCNCs, as displayed in the inset of Figure 4E–G. It is known that the core–shell structure is able to enhance the UCL of the core nanocrystals by suppression of surface quenching effects due to the spatial isolation of the core from the surrounding environmental quenching centers. The observed enhancement here suggests that the epitaxial shell NaYF_4_ is more effective than NaGdF_4_ and NaLuF_4_ in protection of the ultrasmall core against the surrounding quenching process. This is reasonable, as the shell host lattice of NaYF_4_ is identical to the one of the core, thus able to grow a more perfected shell layer onto the ultrasmall core nanocrystals.

To investigate the upconversion mechanism of β-NaYF_4_:Yb, Er@NaLnF_4_ (Ln = Y, Gd, Lu) nanocrystals excited at 980 nm, we acquired the dependence of the UCL intensities at 540 and 659 nm on the pump laser power. The number of photons required to populate the intermediate energy level were calculated based on the formula: *I* ∝ *P^n^*, where *I* is the emission intensity, *P* is the pump laser power, and *n* is the number of photons involved in the excitation process. A double-logarithmic plot was employed to obtain the number of photons, namely, the slope of linear line fitting. As shown in Figure 4H, the numbers of photons (*n*) were determined to be 1.60 ± 0.1, 1.73 ± 0.1, and 1.86 ± 0.1 for the shells of the NaYF_4_, NaGdF_4_, and NaLuF_4_ emissions at 540 nm, respectively. Similarly, the numbers of photons (*n*) were 1.31 ± 0.1, 1.21 ± 0.1, and 1.34 ± 0.1, corresponding to the shells of the NaYF_4_, NaGdF_4_, NaLuF_4_ emissions at 659 nm (see Figure 4I). These results indicate that two photon processes are involved in producing both the 540 and 659 nm UCL emissions, for both large- and small-sized UCNCs [1,28,29]. Figure 4J shows the energy levels of Yb^3+^ and Er^3+^ ions as well as the proposed two-step energy transfer upconversion (ETU) processes [29]. After absorbing excitation photons at 980 nm, the Yb^3+^ ion gets excited from the ground ^2^F_7/2_ state to the exclusive excited ^2^F_5/2_ state. Two successive energy transfers to neighboring Er^3+^ ions excite them from the ground ^4^I_15/2_ state to the ^4^I_11/2_ state, then to the ^4^F_7/2_ state. Nonradiative relaxations from the ^4^F_7/2_ state can populate the lower ^2^H_11/2_ and ^4^S_3/2_ levels, from which UCLs at 525 and 540 nm are produced, respectively. The red UCL at 659 nm originates from the radiative decay from the ^4^F_9/2_ state to the ground state, which can be populated either from nonradiative relaxations from the ^4^S_3/2_ level or the energy transfer from Yb^3+^ ions to the Er^3+^ ion at the ^4^I_13/2_ state. Moreover, the similar slope values of the UCL at both 540 and 659 nm for different types of shell layer indicate that the shell layer does not alter the upconverting mechanism to enhance the UCL of the core, instead, by providing the spatial protection, in line with our aforementioned discussions. The smaller slope values for UCL at 659 nm, compared to those at 540 nm, might originate from the “saturation effect” at the ^4^I_13/2_ state, which is known to have a long lifetime in the order of milliseconds, thus easily enabling the upconverting rate to surpass the decay rate at this state.

## 3. Materials and Methods

### 3.1. Materials

All LnCl_3_·6H_2_O (99.9%, Ln = Y, Yb, Er) and RE_2_O_3_ (RE = Y, Gd, Lu) were obtained from Jianfeng Rare-Earth Limited Company, Conghua, China. The sodium oleate (Na-OA, >97%), sodium trifluoroacetate, and trifluoroacetic acid (TFA, 97%) were purchased from Aladdin (Shanghai, China). Oleic acid (OA), oleylamine (OM), and octadecene (ODE) were obtained from Sigma-Aldrich (Shanghai, China). Other chemical reagents, such as absolute ethyl alcohol and hexane, were acquired from Sinopharm Chemical Reagent Co., Ltd., Beijing, China. All chemicals were used as received without further purification.

### 3.2. Synthesize Rare-Earth Oleates

The rare-earth oleates in this work were prepared by reacting solutions of the corresponding rare-earth chlorides with sodium oleate, modified from a typical synthesis procedure given in the literature [30]. Typically, 1 mmol LnCl_3_·6H_2_O (Ln = Y, Yb, Er and Y^3+^:Yb^3+^:Er^3+^ = (70 − *x*)%:30%:*x*%) and 3 mmol sodium oleate were dissolved in 3 mL of deionized water, 3.5 mL of absolute ethyl alcohol and 7 mL of hexane. The resulting solution was heated to 60 °C and kept at that temperature for 12 h. The transparent organic phase containing the rare earth oleates were obtained after washing the final complex three times with deionized water in a separatory funnel.

### 3.3. Synthesis of β-NaYF_4_:Yb^3+^/Er^3+^ UCNCs

Typically, *x* mmol sodium oleate, 5.2 mL of OA, 5.1 mL of OM and 9 mL of ODE were first mixed with the pre-prepared rare-earth oleates. Then, the temperature was raised up to 100 °C under argon flow and under vigorous stirring to remove water and hexane accompanied with rare-earth oleates. After aging for 60 min, solid ammonium fluoride (4, 5 or 8 mmol) was added into the above solution and vigorously stirred for 30 min at 100 °C. Subsequently, the reaction mixture was heated to 220–300 °C at a rate of 10 K·min^−1^ for half an hour. After naturally cooling down to room temperature, the reaction solution was supplemented with an equal volume of ethanol, and the resulting nanoparticles was collected by centrifugation. The particles were then purified by redispersing the precipitate in 3 mL of hexane, followed by washing with ethanol several times. The final products were dispersed in 10 mL of hexane for further use.

### 3.4. Synthesis of NaYF_4_:Yb^3+^/Er^3+^@NaREF_4_ UCNCs

The core–shell nanoparticles were synthesized using the thermal decomposition method. Typically, 0.5 mmol RE_2_O_3_ (RE = Y, Gd, Lu) was dissolved in a 10 mL trifluoroacetic acid (TFA) aqueous solution with a concentration of 50%. The solution was heated at 90 °C to yield a transparent solution. The shell precursor (CF_3_COO)_3_RE was then obtained by evaporating the solution to dryness under an argon gas purge. Subsequently, 10 mL of OA, 10 mL of ODE, and 5 mL of the pre-prepared β-NaYF_4_:Yb^3+^/Er^3+^ in cyclohexane were added into the three-neck flask, and the mixture was heated at 120 °C for 30 min to distill low-boiling liquid, such as water and hexane. The solution was then heated to 300 °C at a rate of 15 K·min^−1^ under argon protection, and kept at this temperature for 30 min. Lastly, the reaction mixture was cooled to room temperature and the product was collected as described above for the naked core particles. The final products were dispersed in 10 mL hexane for further uses.

### 3.5. Instrumentation

The size and morphology of the β-NaYF_4_:Yb^3+^/Er^3+^ and NaYF_4_:Yb^3+^/Er^3+^@NaREF_4_ core–shell nanocrystals were characterized by transmission electron microscopy (TEM) using a JEOL JEM-2010 microscope (JEOL Ltd., Tokyo, Japan) at an acceleration voltage of 200 kV. Size histograms were derived from TEM images by analyzing the dimensions of a minimum of 150 particles with the software Image J. The powder X-ray diffraction (XRD) patterns were performed with a Siemens D500 diffractometer (Bruker Beijing Scientific Technology Co. Ltd., Beijing, China) using Cu Kα radiation (λ = 0.15418 nm). The 2θ angle of the XRD spectra was recorded at a scanning rate of 5°/min. The UCL spectra were recorded using a spectrometer (Ocean optics FLAME-S-VIS-NIR) under excitation at 980 nm using a fiber-coupled laser diode (BWT Beijing Ltd., Beijing, China). The composition of nanocrystals with different Er^3+^ concentrations was measured by inductively coupled plasma atomic emission spectroscopy analysis (ICP-AES) using the PerkinElmer optical emission spectrometer (Optima 8300) (Shanghai, China).

## 4. Conclusions

We report here on a controlled synthesis of pure hexagonal phase NaYF_4_:Yb/Er nanoparticles with a uniform sub-10-nm size via simple variation of the reaction temperature and the ratio of Na^+^/Ln^3+^/F^−^. The control of reaction temperature enables the production of nanocrystals with a pure hexagonal phase, while a simultaneous control of both the concentrations of NaOA and the NH_4_F precursors entail a dramatic reduction of the nanocrystal size of β-NaYF_4_:Yb^3+^/Er^3+^ to merely 5.4 nm. In addition, the optimum activator concentration of Er^3+^ for a 5.4-nm-sized NaYF_4_:30% Yb/*x*% Er nanocrystals was determined to be about 6.5 mol %, showing a 5-fold higher UCL than that of the canonical formula of β-NaYF_4_:30% Yb/2% Er of the same particle size. Moreover, a set of sub-10-nm NaYF_4_:Yb/Er@NaLnF_4_ (Ln = Y, Gd, Lu) core–shell UCNCs (core size: 5.4 nm; shell thickness: 1.9 nm) have been synthesized, manifesting UCL enhancement of the core by about 85-, 70- and 50-fold, respectively. These sub-10-nm core and core–shell UCNCs with enhanced UCL might find wide biophotonic applications ranging from in vivo bioimaging to biosensing.

## Supplementary Materials

Supplementary Materials are available online. Figure S1: UCL spectra of NaYF_4_:30% Yb/2% Er nanocrystals with different sizes synthesized by using Na^+^, Ln^3+^ and F^−^ with a molar ratio of 8:1:8, 5:1:5, 2.5:1:4, 4:1:4, 6:1:4, respectively. (Reaction temperature, 300 °C); Table S1: Nanocrystal compositions of Na^+^, Y^3+^, Yb^3+^, Er^3+^ as measured by inductive coupled plasma atomic emission spectroscopy (ICP-AES).
