# Supplementary material for: Controlled Synthesis of Monodisperse Hexagonal NaYF4:Yb/Er Nanocrystals with Ultrasmall Size and Enhanced Upconversion Luminescence
Source: Molecules. 2017 Dec 1;22(12):2113. doi: 10.3390/molecules22122113 (PMC6150031; doi:10.3390/molecules22122113)
Supplement: Supplementary file 1 [file molecules-22-02113-s001.pdf]

Supplementary Materials for

Controlled synthesis of monodisperse hexagonal $NaYF_4$:Yb/Er nanocrystals with ultrasmall size and enhanced upconversion luminescence

Hui Li , Lei Xu and Guanying Chen*

MIIT Key Laboratory of Critical Materials Technology for New Energy Conversion and Storage, School of Chemistry and Chemical Engineering & Key Laboratory of Micro-systems and Micro-Structures, Ministry of Education, Harbin Institute of Technology, Harbin 150001, China; huili@hit.edu.cn (H.L.); xulei82@hit.edu.cn (L. X.) Correspondence: chenguanying@hit.edu.cn (G.C.)

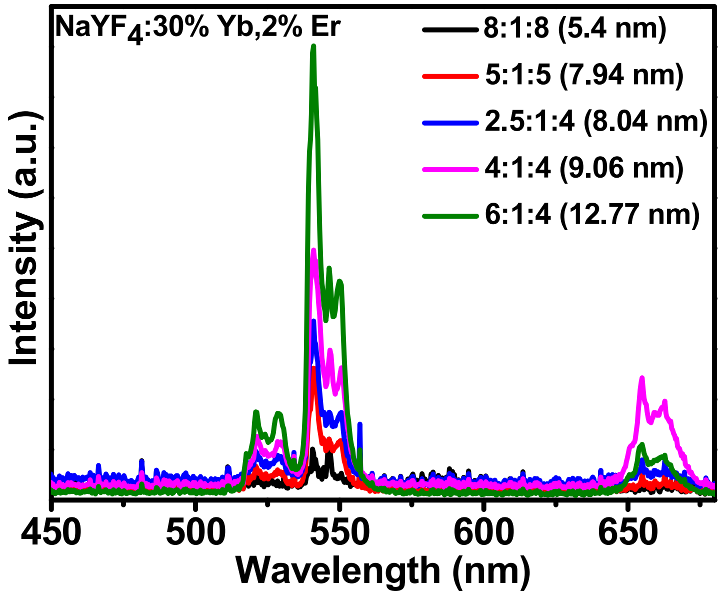

Figure S1. The upconversioin luminescence (UCL) spectra of $NaYF_4$: 30% Yb,2% Er nanocrystals with different sizes synthesized by using Na^+, $Ln^{3+}$and F^- with a molar ratio of 8:1:8, 5:1:5, 2.5:1:4, 4:1:4, 6:1:4,respectively. (Reaction temperature, 300°C)

Table S1. Nanocrystals composition of Na^{+}, Y^{3+}, Yb^{3+}, Er^{3+} as measured by inductive coupled plasma atomic emission spectroscopy (ICP-AES) after dissolving the nanoparticles in diluted HNO_3 solution.

Designed NCs	Concentration of cation ions of NCs measured by ICP-AES				Calculated molar ratio		
	Na^{+}(m mol/L)	Y^{3+}(m mol/L)	Yb^{3+}(m mol/L)	Er^{3+}(m mol/L)	Er^{3+}/(Yb^{3+} +Y^{3+}+Er^{3+})	Yb^{3+}/(Yb^{3+} +Y^{3+}+Er^{3+})	Yb^{3+} /Er^{3+}
NaYF4:30% Yb,2%Er	0.3568	0.1257	0.0477	0.0037	2%	27%	12.8
NaYF4:30% Yb,5%Er	0.1638	0.0532	0.0196	0.0038	5%	26%	5.1
NaYF4:30% Yb,10%Er	0.2422	0.0952	0.0406	0.0094	6.5%	28%	4.3
NaYF4:30% Yb,12%Er	0.1415	0.0348	0.0156	0.0069	12%	27%	2.3
NaYF4:30% Yb,15%Er	0.2132	0.1077	0.0441	0.0238	14%	25%	1.9
